# Wide Dynamic Range Digital Aberration Measurement and Fast Anterior-Segment OCT Imaging †

**DOI:** 10.3390/s24165161

**Published:** 2024-08-10

**Authors:** Mengyuan Ke, Abhishek Kumar, Thor E. Ansbæk, Rainer A. Leitgeb

**Affiliations:** 1 Center for Medical Physics and Biomedical Engineering, Medical University of Vienna, 1090 Wien, Austria; mengyuan.ke@meduniwien.ac.at; 2 Wavesense Engineering GmbH, 1190 Wien, Austria; abhishek.kumar@wavesenseengineering.com; 3 OCTLIGHT ApS, 2800 Lyngby-Taarbæk, Denmark; thor@octlight.com

**Keywords:** sensorless aberrometry, optical coherence tomography, dual-modality, MHz laser, corneal imaging

## Abstract

Ocular aberrometry with a wide dynamic range for assessing vision performance and anterior segment imaging that provides anatomical details of the eye are both essential for vision research and clinical applications. Defocus error is a major limitation of digital wavefront aberrometry (DWA), as the blurring of the detected point spread function (PSF) significantly reduces the signal-to-noise ratio (SNR) beyond the ±3 D range. With the aid of Badal-like precompensation of defocus, the dynamic defocus range of the captured aberrated PSFs can be effectively extended. We demonstrate a dual-modality MHz VCSEL-based swept-source OCT (SS-OCT) system with easy switching between DWA and OCT imaging modes. The system is capable of measuring aberrations with defocus dynamic range of 20 D as well as providing fast anatomical imaging of the anterior segment at an A-scan rate of 1.6 MHz.

## 1. Introduction

Optical coherence tomography (OCT) has acquired popularity as a well-established and non-invasive imaging modality in biomedical applications, especially in ophthalmology for clinical diagnosis [1]. OCT measures the complex electric field of backscattered light, with cross-sectional images generated by the transverse scanning of the incident beam from measurement of echo time delay, and volumetric data generated by sequential recordings of cross-sectional images. The decoupled nature of its axial (1–15 µm) [2] and lateral resolution enables depth-resolved volumetric structural information with high axial resolution. Cellular resolution at the ocular fundus has been achieved when hardware-based adaptive optics are incorporated [3]. Ever since the introduction and first applications of Fourier domain OCT, the speed of OCT has been much advanced by orders of magnitudes due to its sensitivity advantage [4,5] compared to the original time domain OCT. The development of high-speed lasers further exploits the advantage of MHz sweep rate with phase stability for an expanded field of view (FOV) [6,7,8,9]. Commercialized OCT has been used for cornea tomography in clinical settings. The main challenge of anterior segment imaging is to achieve a wide lateral imaging range with sufficient imaging depth while maintaining a high volume rate. Regarding the depth ranging capability, spectrometer-based (SD)-OCT such as Cirrus HD-OCT (version 8) is limited to 2 mm depth unless the conjugated mirror image is used and stitched in postprocessing to extend the depth range. Thereby, extended SD-OCT systems reported a maximum wide scan up to 15.5 mm laterally and 5.8 mm in depth. SS-OCT with the speed advancement up to MHz spectral rates and typical center wavelengths of ~1 µm or ~1.3 µm has distinct advantages over spectrometer-based OCT in terms of speed and signal roll-off with depth. SS-OCT such as Triton OCT (Topcon Corporation, Tokyo, Japan) and CASIA SS-1000 OCT (Tomey Corporation, Nagoya, Japan) (both at 1310 nm) have reported 6 mm and 3 mm imaging depths, respectively. The performer requires an external add-on lens to be attached to the system in order to switch from posterior imaging to anterior imaging, whereas the latter is limited to a speed of 100 kHz. Various commercially available OCT systems for anterior segment imaging have been well summarized [10]. Longer wavelengths experience less scattering, but water absorption becomes dominating, especially for ~1300 nm. Thus, an SS-OCT with a fast sweeping rate and a wavelength in a trade-off zone is desirable for anterior ocular imaging.

To achieve multi-MHz imaging speed, frequency-swept lasers operating in Fourier domain mode locking(FDML) [11,12], frequency comb sources with stretched-pulse mode locking (SPML) [13], or micro-electromechanical system-supported vertical cavity surface-emitting lasers (MEMS-VCSEL) [14,15,16] have been introduced in OCT applications. The latter, with its advantages in single-mode operation, long-range coherence, semiconductor wafer batch manufacturing for mass production, and adjustable axial scan rate for variable scanning protocols, is a desired candidate source for commercialized systems.

A fast imaging rate is a prerequisite for phase-sensitive functional extensions of OCT applied to the human eye in vivo. Previously, our group demonstrated that swept-source OCT (SS-OCT) can be employed for quantitative aberrometry with a guide star-based digital lateral shearing (DAO-DLS) technique [17]. The approach of digital aberrometry assesses the wavefront error (WFE) at the pupil plane directly from the complex point spread function (PSF) in the retinal plane, which can be straightforwardly combined with OCT imaging and requires no prior knowledge of the parameters of the system. However, the dynamic range is limited to within ±3 D due to a relatively small FOV because of the limited sweep rate of the laser (450 kHz) and maximum mechanical scanning frequency of the non-resonant galvanometric scanner [18].

For the present work, we employed a 1.6 MHz electrically pumped vertical-cavity surface-emitting laser (VCSEL) swept source (OCTLIGHT). The fiber-based system adopts easy switching between an MHz OCT channel and a digital wavefront aberrometry (DWA) channel, making it a dual-modality system capable of fast OCT imaging and aberration measurement. By incorporating a Badal-like defocus compensation in the design, we further expand the dynamic measurement range of defocus up to 20 D. We demonstrate the proof in principle of this dual-modality system in a model eye. Such a system capable of providing both refractive errors and corneal anatomic information would be desired in clinical care, especially for precise treatment planning of refractive error-related eye diseases.

## 2. Materials and Methods

### 2.1. Dual-Mode System

**Figure 1:**
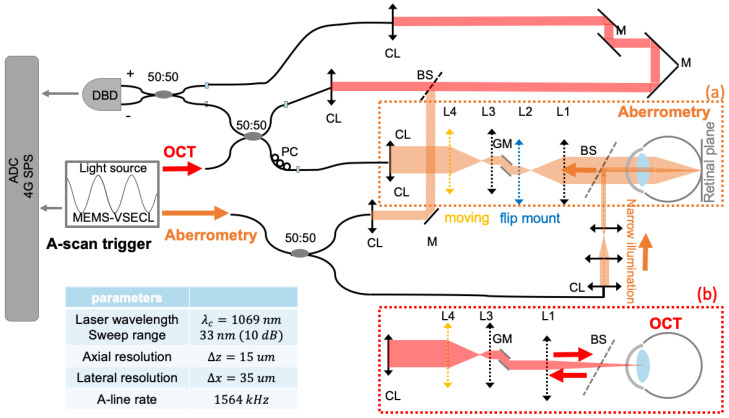
Dual-modality digital aberrometry swept-source optical coherence tomography. Focal length of L1: f = 100 mm, L2: f = 30 mm, L3 = 25 mm, L4 = 100 mm. PC: polarization controller, M: Mirror, CL: collimator, BS: non-polarizing beam splitter, ADC: analog to digital converter. Orange dotted box: sample optical path in aberrometry. Red dotted box: sample optical path in OCT. (**a**) In aberrometry mode, a narrow pencil beam (indicated by an orange color and arrow) illuminates the retina; backscattered light (indicated by a red color and arrow) exits the pupil plane and is then de-scanned on its way back before detection and a single-pass volumetric PSF is acquired. (**b**) In OCT mode, light (indicated by a red color and arrow) is focused on and scanned across the anterior segment, with backscattered light being detected along the same path.

The dual-mode system depicted in Figure 1 is designed to switch between swept-source OCT (SS-OCT) and digital wavefront aberrometry (DWA) while sharing the same source and detection schemes. For the fiber-based SS-OCT, an electrically pumped VCSEL swept-source (OCTLIGHT) with a center wavelength of 1069 nm, bandwidth of 33 nm, and sweep rate of 1.6 MHz is used, providing an axial resolution of ~15 µm in tissue. The system has a lateral resolution of 35 µm in air and a sensitivity of ~98 dB. Tomographic scans are acquired at a B-scan rate of 640 Hz. Both forward and backward sweeps are utilized to profit from the maximum available sweep rate. In DWA mode, a narrow (0.6 mm diameter) beam is used for illumination, as is done in conventional clinical aberrometry; this acts as a pseudo-point source that reflects back the light passing through the full pupil of the eye, capturing the single-path optical aberrations. The narrow beam suffers fewer aberrations and can form a diffraction-limited spot on the retina; in addition, the depth of focus (7.74 mm) for such a narrow beam is quite large and there is no tight focus on the retina, alleviating the need for fine focus adjustment. The back-reflected light passes via the optics and scanners of the OCT sample arm before being focused and descanned at the detection fiber plane. The detection and standard OCT processing lead to volumetric PSFs, which are post-processed using digital lateral shearing-based DLS-DAO to reconstruct the wavefront error as in a previous publication by our group [17]. Lens L4 in the sample arm (Figure 1) is placed on a translational stage, similar to a Badal optometer [19], to compensate for the defocus error over a wide range before detection. For alignment and adjustment of focus correction by L4, a cross-sectional tomogram through the PSF is processed and displayed in real-time. In SS-OCT anterior chamber imaging mode, L2 is flipped out from the optical path to focus the beam within the anterior segment of the eye. The light is scanned across the anterior segment, and backscattered light is detected along the same path in a double-pass configuration. OCT anterior segment and DWA signals are detected by a shared dual-balanced photodetector (DBD) with a 2.5 GHz radio frequency (RF) bandwidth (PDB482C-AC, Thorlabs Inc., Newton, NJ, USA). The signals are digitized by a high-speed 12-bit analog-to-digital converter (ADC) at a speed of 4 Giga samples per second (GSPS) (ATS9373, Alazar Technologies Inc., Pointe-Claire, QC, USA).

### 2.2. Bidirectional Sweeps

**Figure 2:**
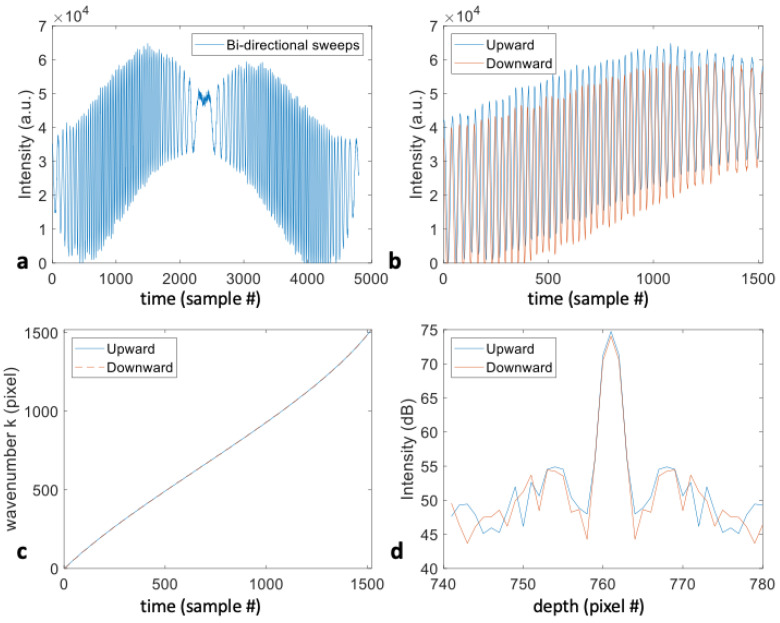
(**a**) Full bidirectional sweeps; (**b**) upward and downward unidirectional sweep; (**c**) time needed for the wavenumber k-remapping function generated from a single reflector to evenly sample the sweeps in k space; (**d**) FFT of upward and downward unidirectional sweep after k-remapping, resulting in uniform k-space. The X-axes are the analog-digital-converter (ADC) samples, which are related to a wavelength sweep over time.

For source sweep characterization, we utilized the bidirectional sweeps of the 1.6 MHz source and measured a single mirror interface as the sample. From Figure 2b, a similar shape of the spectral interference pattern can be observed for the upward and downward sweep in the electrically pumped VCSEL laser. In the following analysis, we manually cut the bidirectional sweeps based on a single reflector interference signal and independently process the unidirectional sweeps to achieve the best axial resolution performance. It is inevitable that the internal mechanical motions of the sweeping mechanism may produce operational instability in the time-frequency relation. A Mach–Zehnder interferometer (MZI) can be used as a timing reference to acquire the spectrum simultaneously with imaging in order to correct the sweep-to-sweep variation; however, this solution is expensive in terms of both computation and memory, requires two-channel analog-digital-converter (ADC) acquisition, and is prone to clocking glitches and sampling errors above 1.3 GSPS [16]. Instead, we use the previously measured interference signal from a single reflector and use the generated k-remapping function to correct the nonlinearity of the subsequently acquired sample spectra. The k-rescaling functions for both directional sweeps are further optimized to compensate for system dispersion, resulting in similar axial best resolution performance, as seen in Figure 2d. Utilizing both sweeps allows the full speed of the laser to be exploited.

### 2.3. Sample for Imaging

To validate the MHz VCSEL source application in DWA and test the approach involving extended defocus error dynamic range, an artificial eye (Skia/Retinoscope Trainer, Heine Optotechnik GmbH; Herrsching, Germany) with an adjustable pupil size of 4–6 mm, focal length of 32 mm, and set defocus error ranging over 12 D was used as the sample. Additional trial lens sets of defocus error over 8 D were used and placed in front of the model eye to extend the introduced defocus over a range of 20 D. For cylinder error measurements, trial cylinder lenses of −3 D and 0.25 D were placed in front of the model eye. Volumetric PSFs were acquired at 5 mm pupil diameter of the model eye for the defocus range from −4 D to +6 D and at 4 mm pupil diameter for the defocus range from +7 D to +15 D. The pupil size had to be reduced for larger defocus error ranges in order to fit the detected beam size to the limiting scanner mirror diameters. To test the performance of the OCT, the anterior segment image of a model eye (Rowe model eye) was recorded at a 1.6 MHz A-scan rate. Volume data consisting of 4800 × 2000 × 400 voxels (spectral × fast axis × slow axis sampling voxels) were acquired with oversampling along the fast axis due to the speed limitation imposed by the galvo-scanners. Due to data size limitations for wide-field 3D imaging, the number of recorded spectra along the fast axis was down-sampled by a factor of 10.

### 2.4. Postprocessing

For aberration analysis in DWA, the volumetric PSFs were used to calculate the WFE using the previously published DLS-DAO algorithm [17]. In brief, an enface PSF is chosen at a selected depth corresponding to the retinal plane from the complex volumetric data. The 2D Fourier transform of the complex-valued PSF image yields the pupil plane. The slope per pixel within the circular pupil is calculated using the phase differences between the calculated pupil field and its digital copies translated by one pixel along the horizontal and vertical directions in the pupil plane, respectively. The WFE is computed by fitting the derivative of the Zernike polynomials up to the 6th order. Defocus and cylinder error are obtained from the Zernike coefficients of the relevant polynomial terms according to the ANSI standard [20]. For defocused PSFs, we corrected the pupil aspect ratio introduced by the nonconjugate scanners for the orthogonal lateral directions (Appendix A.2). To demonstrate the DWS for the defocus and cylinder error measurements, we further correct the PSFs with obtained wavefront aberrations. This is accomplished by multiplying the conjugate phase errors reconstructed from the obtained wavefront as phasors with the complex-valued aberrated image in the Fourier plane. Details can be found in our previous publication [17]. For corneal imaging analysis, each upward and downward sweep undergoes the standard OCT processing pipeline independently, i.e., background subtraction, wavenumber (k-) linearization, spectral reshaping, Fourier transformation, and log scaling via a customized written script (Matlab, 2023a).

## 3. Results

**Figure 3:**
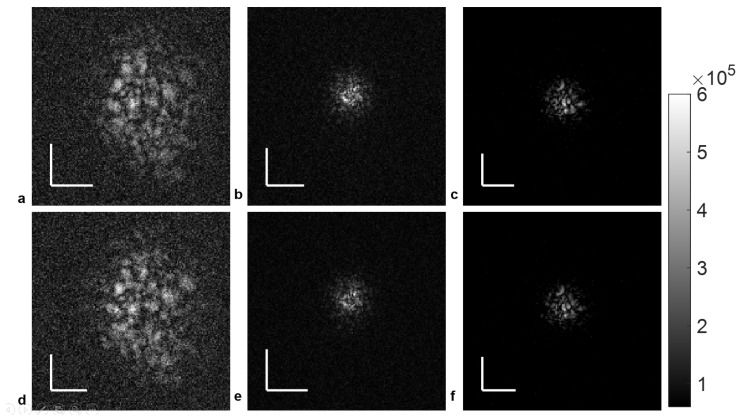
(**a**) +3 D defocus error en face PSFs imaged before defocus compensation for upward sweep; (**b**) +3 D defocus error en face PSFs imaged after defocus compensation for upward sweep; (**c**) 0 D reference PSF imaged using upward sweep; (**d**) +3 D defocus error en face PSFs imaged before defocus compensation for downward sweep; (**e**) +3 D defocus error en face PSFs imaged after defocus compensation for downward sweep; (**f**) 0 D reference PSF imaged using downward sweep. Scale bar: 0.1 mm.

For demonstration of the measurement principle, volumetric PSFs of the model eye were acquired with 5 mm pupil diameter and +3 D defocus. Because we cannot assume a priori that upward and downward laser sweeps that are processed independently will yield the same aberration measurement, it is necessary first to treat both independently, and then perform a careful quantitative comparison of their results. Figure 3a–c shows the results using only the upward laser sweeps, first without defocus precompensation (Figure 3a) and then with precompensation using the Badal-like principle via translation of lens L4 (Figure 3b). Figure 3d–f shows the analog results for the downward laser sweeps. The results compare well to reference measurements taken with the 0 D setting of the model eye, depicted in Figure 3c,f for upward and downward sweeps, respectively. Already, it can be qualitatively observed that the upward and downward sweeps of the electrically pumped MEMS-VCSEL laser in Figure 3 show similar PSFs. The defocus error of +3 D, which was difficult to measure without defocus correction due to the large PSF extension, is now effectively minimized before detection, resulting in comparable PSFs to the 0 D case.

### 3.1. Defocus Error PSF Measurements

**Figure 4:**
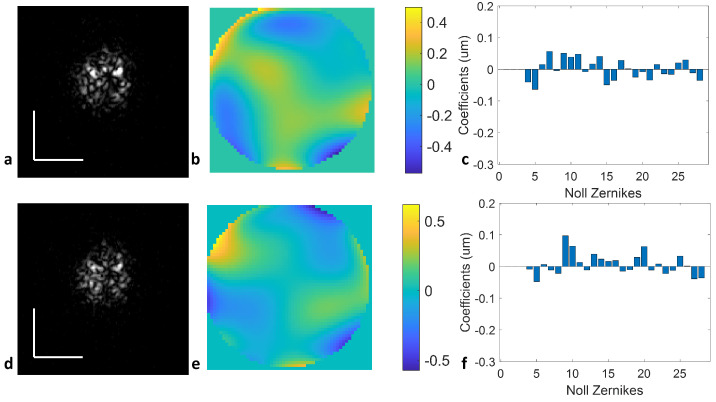
(**a**) −4 D defocus error en face PSF after defocus compensation for upward sweep; (**b**) calculated residual WFE in microns using DAO-DLS for upward sweep; (**c**) bar plots of Zernike coefficients for upward sweep; (**d**) −4 D defocus error en face PSF after defocus compensation for downward sweep; (**e**) calculated residual WFE in microns using DAO-DLS for downward sweep; (**f**) bar plot of Zernike coefficients for downward sweep. Scale bar: 0.1 mm.

For the next case, volumetric PSFs were acquired from the model eye with a 5 mm pupil diameter and +4 D defocus error while applying defocus error pre-compensation. The extracted PSF images were used to calculate the WFE and the residual Zernike coefficients using DAO-DLS. The peak-to-valley residual WFE results in Figure 4b,e indicate that the predominating defocus error has been eliminated. Using the upward and downward sweeps independently results in similar residual aberrations, described by the root mean square wavefront error (RMSWFE) as 0.1643 µm and 0.1711 µm, respectively. Both values are well below the Rayleigh criterion, as seen in the bar plots of Zernike coefficients (Figure 4c for the upward sweep and Figure 4f for the downward sweep). This provides quantitative substantiation, indicating that both upward and downward sweeps yield highly precise wavefront error measurements. Taking the averaged phase map calculated in Figure 4b,e, the calculated RMSWFE is further reduced to 0.1433 µm, the residual defocus to 0.027 D, and the cylinder error to −0.089 D. This is due to the averaging of the two independent sweeps minimized the phase fluctuation, which reduces the residual RMSWFE after Badal lens correction.

Volumetric PSFs were acquired at a 4–5 mm pupil diameter of the model eye for the range of −5 D to +15 D defocus, with the defocus error precompensated. Selected en face PSFs for upward sweep images are shown here with the pupil plane aspect ratio corrected for each diopter case other than 0 D. The details of correction are provided in Appendix A.2. The defocused PSF > 7D appears larger due to the oversampling step size and focusing errors introduced by manually placing the trial lens in front of the model eye. The former was due to the scanning mirrors; detailed explanations can be found in Appendix A.1.

**Figure 5:**
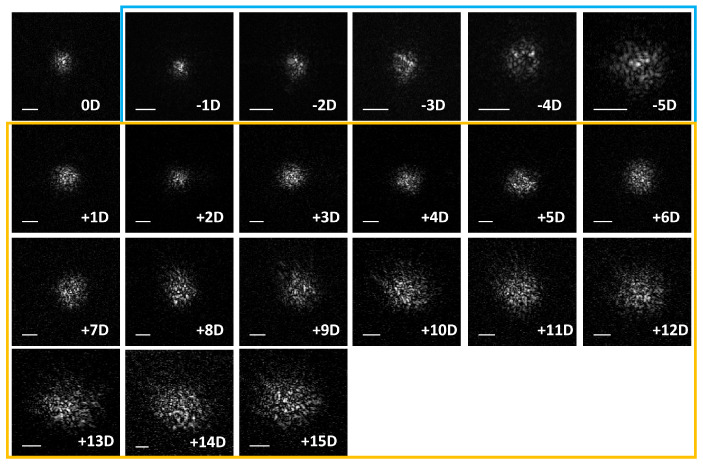
En face PSFs imaged after defocus compensation over a range of 20 D. Blue box: −1 to −5 D, yellow box: +1 to +15 D. Scale bar: 0.1 mm.

Defocus error is a major limitation of DWA, as it causes the PSF to blur, resulting in a significantly reduced SNR of the detected OCT signal of the PSF beyond the ±3 D range, as previously reported in [18]. By precompensating the defocus via translation of lens L4, similar to a clinical Badal Optometer, the defocus is alleviated prior to the detection collimator. With this approach, we demonstrate focused PSF imaging in the DWA mode over an extended defocus dynamic range of up to 20 D, as seen in Figure 5. En face PSFs between −5 D to +6 D shows a similar radial extent compared to the 0 D reference after pre-defocus compensation via translating the L4 lens. The apparently larger en face PSF can be attributed to the oversampling of the highly defocused PSF. Because the model eye defocus range setting was limited, we had to extend the introduced defocus range by placing trial lenses in front of the model eye manually, which may have caused inaccuracies in the introduced defocus.

**Figure 6:**
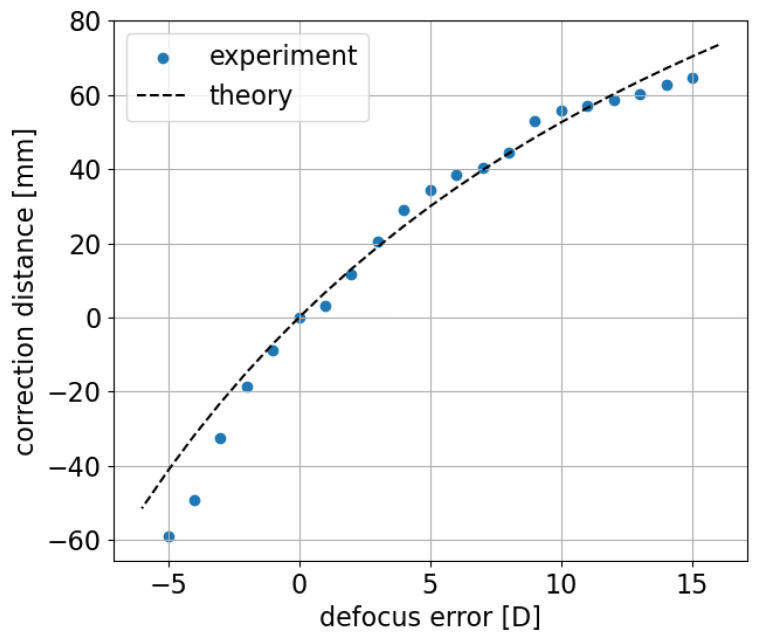
Measured defocus error over a range of 20 D vs. lens L4 translation distance from 0 D.

Nevertheless, this approach to defocus compensation requires no additional elements, only placing the existing lens L4 on a digitally controlled translational stage. We tracked the translation distance of lens L4 for the corresponding defocus error over the range of 20 D with respect to 0 D PSF; the results are compared to the simulated results in Figure 6 on a paraxial approximation of the sample arm optics. The details of the simulation can be found in Appendix A.1.

The displacement of L4 from the 0 D position as a function of compensated defocus (Figure 6) is shown to cover the range of −5 D to +15 D. The experimentally measured relationship of the lens diopter and the required translation distance are similar to the usage of the Badal lens in a Badal optometer [19] and is in good agreement with the paraxial approximation-based simulation for most of the range. The tendency of under-correction for the introduced defocus below −3 D could be due, on the one hand, to the subjective judgment of compensation based on a live cross-sectional speckled PSF image during acquisition. This subjective error can be avoided by careful calibration of the displacement of the Badal lens L4 and an indication of the related defocus precompensation, as is standard in commercial fundus imaging systems. On the other hand, the simulation itself is based on a thin lens approach under paraxial approximation, which may not remain valid for large defocus values.

### 3.2. Cylinder Error Measurements

**Figure 7:**
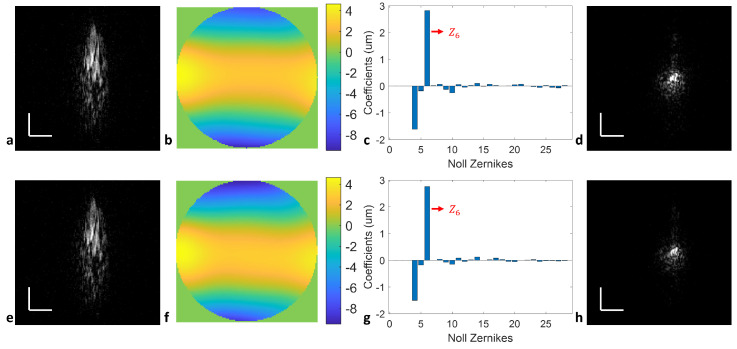
DWA results using the DLS-DAO algorithm for the (**a**) measured cylinder error of −3 D en face PSF in the upward sweep, (**b**) calculated wavefront error map in the upward sweep, (**c**) calculated Zernike coefficients in the upward sweep, (**d**) digitally refocused PSF in the upward sweep, (**e**) measured cylinder error of −3 D en face PSF in the downward sweep, (**f**) calculated wavefront error map in the downward sweep, (**g**) calculated Zernike coefficients in the downward sweep, and (**h**) digitally refocused PSF in the downward sweep. $Z_{6}$: primary vertical cylinder. Scale bar: 0.1 mm.

We performed a further proof-of-concept analysis of the DWA system to measure the cylinder error. Trial cylinder lenses of −3 D and −0.25 D was placed in front of the model eye at 6 mm pupil diameter with the cylinder axis aligned vertically with respect to the optical axis of the model. Both the upward sweeps in Figure 7a–c and the downward sweeps in Figure 7e–g resulted in similar cylinder error measurements of the measured en face PSFs, the calculated wavefront error, and the corresponding Zernike coefficients. For the −3 D trial cylinder lens, the measured cylinder error was −3.02 D at 88.3° and 3.17 D at 88.5° from $Z_{6}$ (the primary vertical astigmatism) using the upward and downward sweeps of the VCSEL laser, respectively. The average of two sweeps provides a cylinder error measurement of 3.1 D at 88.4°. With the calculated WFE, the −3 D cylinder error PSFs can be digitally refocused, as shown in Figure 7d,h.

**Figure 8:**
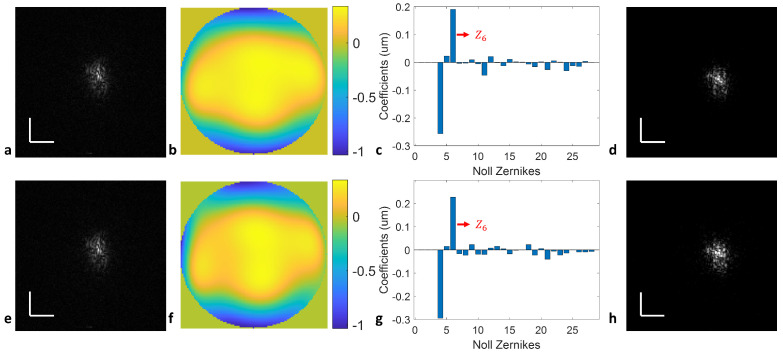
DWA results using the DLS-DAO algorithm for determining a cylinder error of −0.25 D: (**a**) en face PSF of the upward sweep; (**b**) calculated wavefront error map; (**c**) calculated Zernike coefficients; (**d**) digitally refocused PSF; (**e**) en face PSF of the downward sweep; (**f**) calculated wavefront error map using the downward sweep; (**g**) calculated Zernike coefficients; (**h**) digitally refocused PSF. $Z_{6}$: primary vertical cylinder. Scale bar: 0.1 mm.

We further demonstrate that DLS-DAO can detect a cylinder as small as −0.25 D, as seen in Figure 8. The measured cylinder error was −0.227 D at 91.9° and −0.249 D at 91.5° using upward (Figure 8a–c) and downward (Figure 8e–g) sweeps, respectively. The average of the two sweeps provides a cylinder error measurement of −0.238 D at 91.7°. With the calculated WFE, the −0.25 D cylinder error PSFs can be digitally refocused, as shown in Figure 8d,h.

### 3.3. MHz OCT Measurements

**Figure 9:**
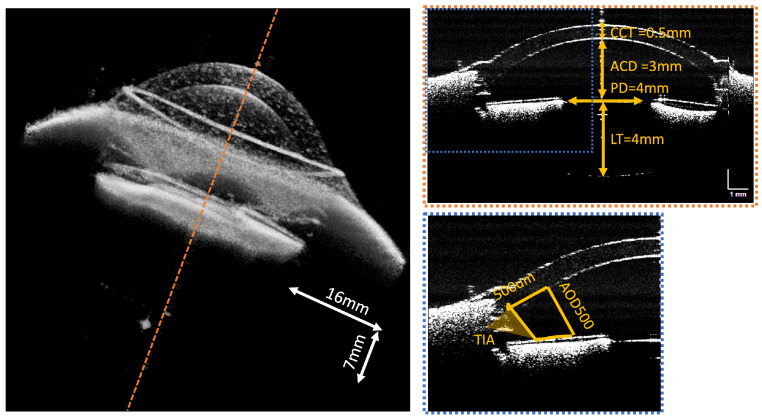
OCT volume reconstructed from the average of upward and downward sweeps, showing sample B-scan (selected at dotted orange line position) magnified at the dotted blue region of the anterior chamber of the model eye recorded at a 1.6 MHz A-scan rate. CCT: central cornea thickness, ACD: anterior chamber depth, PD: pupil diameter, LT: lens thickness, TIA: trabecular-iris area, AOD500: the angle-opening distance at 500 µm anterior to the scleral spur.

**Figure 10:**
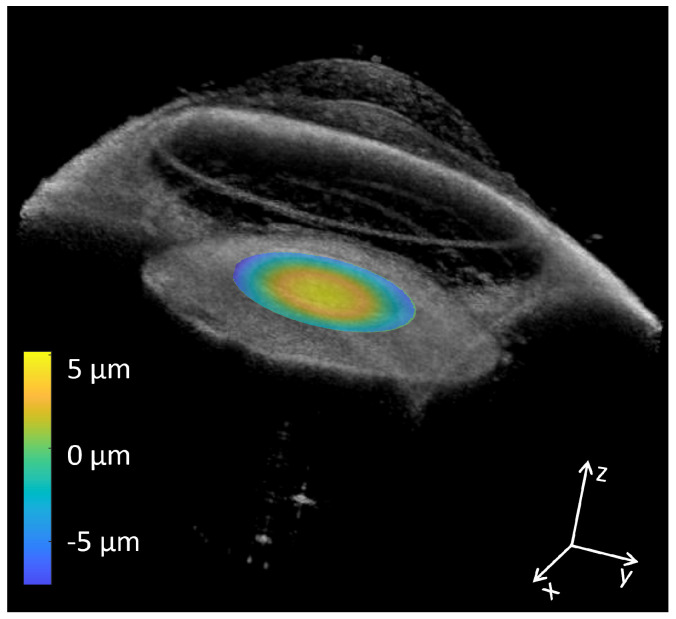
Representative 3D volume rendering of the measured full anterior segment (spectral z ×fast axis x ×slow axis y) showcasing detailed anatomical structures and 2D wavefront map with −3 D defocus error overlaid in the pupil plane illustrating the measured aberrations.

In the OCT anterior segment imaging mode, a 16 mm × 16 mm × 7 mm volume of the anterior chamber of a model eye with known dimensions is acquired at an A-scan rate of 1.6 MHz. Different layers and structures of the anterior segment, such as the anterior cornea, posterior cornea, iris, anterior lens, and posterior lens, can be clearly visualized in the reconstructed volume and B-scan OCT images with a wide field of view in Figure 9. Volumetric rendering clearly shows the layer structures in a rectangular volume. Each directional sweep can be found in the supplementary video (Supplementary Video S1: upward sweep imaging; Supplementary Video S2: downward sweep imaging). All of the gathered information can finally be combined into an integrated visualization, exemplifying the ability of our system to deliver both visual function and structural characterization of the eye (Figure 10).

## 4. Discussion

Stand-alone DWA based on SS-OCT has been previously demonstrated in vivo by our group for measuring ocular aberrations with accuracy comparable to a commercial Hartmann–Shack aberrometer [18]. However, the dynamic range for defocus error detection is limited to ±3 D. Higher defocus diopter errors cause the PSF to blur and increase in size, which cannot be captured by the limited small scanned FOV. Furthermore, PSF blurring results in the loss of signal coupling efficiency into the single-mode detection fiber and causes a significant drop in SNR beyond ±3 D of defocus error. Here, we demonstrate a modified dual-modality SS-OCT system based on a modular design that shares the same light source and detection channel and allows for easy switching between DWA with an extended defocus dynamic range of 20 D and fast anterior segment imaging. Such a multimodal system can be employed for intrasurgical OCT settings, combining the advantages of structural and functional assessment of optical performance for improved refractive surgery outcomes. This pilot study demonstrates that functional aberration measurements can be obtained at a low cost with modifications to existing SS-OCT systems. We expect this quasi-simultaneous assessment of structure and function to enable new avenues for vision research.

By employing a high sweep rate laser (1.6 MHz unidirectional) instead of the previous unidirectional sweep of 450 kHz, it is possible to acquire a larger FOV with sufficient A-scan sampling and without sacrificing the B-scan rate (640 Hz). In particular, the approach in this paper benefits from the concept of a Badal optometer by translating one of the sample arm lenses (L4) with micrometer precision to pre-compensate the dominant defocus term in ocular aberrations, thereby further extending the defocus dynamic range up 20 D. We have demonstrated high wavefront sensitivity of DWA by measuring a cylinder error as small as −0.25 D. We were able to fully exploit the high A-scan rate of 1.6 MHz, as the upward and downward sweeps had similar accuracy regarding the DWA. With independent k-linearization and optimization for each directional sweep, it is possible to effectively double the A-line rate without the need for phase stabilization such as a Fiber Bragg grating (FBG) [21]. By utilizing the bidirectional sweeps rather than discarding the downward sweep, volumetric image data covering the full anterior chamber can be acquired down to the posterior lens and recorded at a full sweep rate of 1.6 MHz. This MHz-speed imaging contributes to improved anterior imaging with low motion distortions compared to typical clinical SS-OCT at the speed of hundreds of kHz A-line rate and is potentially clinically advantageous in terms of patient comfort and improved diagnostics. The advantage of full-speed exploration by averaging the two independent sweeps improves the SNR in imaging and further suppresses the phase fluctuation in wavefront measurement.

Despite the relatively low bandwidth of the electrically pumped VCSEL of ~33 nm, our resulting SS-OCT axial resolution of ~15 µm is comparable to existing commercial anterior segment imaging devices used in the clinics, such as the IOLMaster700 of Carl Zeiss Meditec with axial resolution of 22 µm, the Slit Lamp OCT of Heidelberg Engineering with axial resolution of 25 µm, and the Visante OCT from Carl Zeiss with axial resolution of 18 µm [10,22].

Combining anterior segment imaging with aberrometry is beneficial in refractive error-related ophthalmic surgery. A recent study integrating anterior segment (AS)-OCT with a Hartmann–Shack aberrometer using a defocused beam demonstrated a wide dynamic range of −17 D to + 25 D [23]. Despite the compact design in the study using a shared beam, the SD-OCT at a center wavelength of 840 nm has the limitations of a slow A-scan rate (20 kHz) and sensitivity roll-off that are not ideal for volumetric anterior segment imaging. On the other hand, in our system, the full speed of a 1.6 MHz swept laser centered at 1069 nm facilitates faster and extended anterior imaging in depth.

A change of SD-OCT to SS-OCT may help with SNR roll-off to extend the imaging depth as well as the imaging speed. However, the typical wavelength of swept source lasers is around 1 µm, which is disadvantageous for the mostly silica-based area detectors used in Hartmann–Shack sensors. Their use of a shared wavelength beam requires additional modification of beam geometry; a large retinal spot size of 280–375 µm makes it difficult to determine the centroid in the Hartmann–Shack aberrometer, requiring large pitch size, which leads to a trade-off between sensitivity and dynamic range. In our system, we share the same swept source laser centered at 1069 nm between OCT and DWA with reduced power. In DWA, a diffraction-limited stationary spot of 67 µm is formed at the retina by a narrow illumination beam. The reflected light then passes through the full pupil of the eye, capturing the single-path optical aberrations. Therefore, our aberration analysis is theoretically tolerant to the cancellation of odd aberrations thanks to the second pass through the optics [24,25]. The DWA with DLS-DAO approach to aberration analysis further simplifies the hardware requirements, reduces costs, and demonstrates high sensitivity by detecting cylinder errors as small as -0.25 D. The measurement sensitivity of DLS-DAO depends on the pixel pitch in the pupil plane and the number of pixels in the pupil/phase plane. A densely sampled larger FOV-measured PSF can offer a high dynamic range and precise wavefront error analysis [17]. The measurement range in DWA is limited by (1) the physical translation distance of lens L4 and (2) the alignment during movement. A further range extension may be demonstrated with an even larger traveling range of lens L4 in the system using tailored rather than off-the-shelf optomechanical components. The alignment of the beam exiting the model eye towards the detection collimator during displacement of L4 is well maintained by a micrometer-precision digitally controlled stage to ensure reproducible repositioning and alignment.

Despite the widespread popularity of Hartmann–Shack aberrometers as wavefront sensors in vision science, DWA paves the way for new sensorless aberrometers for future clinical care thanks to its direct access to the PSF at the retina plane, straightforward combination with standard OCT, high sensitivity, and wide dynamic range.

However, other limitations are present in the current design. We performed defocus precompensation after the galvo-scanner mirrors, which imposes pupil shape asymmetry due to the 1 cm separation of the scanner mirror pairs. This requires additional processing steps to correct the uneven scanning image step sizes imposed by the asymmetry, as outlined in Appendix A.2. A more ideal design would be to incorporate this defocus compensation method before the galvo-scanner mirrors; in this way, the beam is collimated and has the same diameter at the two galvo-scanner mirrors, which ensures that the scanning step size at the fiber detection plane remains the same along the vertical and horizontal axes. Alternatively, the two scanners could be separated and optically conjugated using relay lenses. Although we currently only correct the defocus errors, a liquid crystal device [26] or a cylindrical lens could be implemented in the future to extend the dynamics for cylinder error measurements. Nevertheless, the dynamic range of defocus error after proper correction is comparable to commercially available aberrometers. This DWA approach has been previously validated in human subjects by our group [17,18] to accurately measure 2nd-order aberrations. Other future work to improve this study could include replacing the current galvo-scanners with resonant scanners to speed up the B-scan and volume rate as a way to fully exploit the MHz speed swept laser. Future investigation of the biometrics of the anterior segment in the volumetric scan would also be of interest for research in eye modeling, cataract and glaucoma diagnostics, as well as applications such as contact lens fitting and intraocular lens design.

## 5. Conclusions

We have demonstrated a customized dual-mode OCT system based on an electrically pumped MHz VCSEL swept source at a 1.6 MHz A-scan rate. The system can perform DWA with a wide defocus error dynamic range of 20 D and fast anterior chamber OCT volumetric imaging with full-speed utilization. The availability of both visual performance and anatomical information (Figure 10) may aid in the precise personalized planning of refractive error surgery and treatment of refraction-related eye diseases.

## Figures and Tables

**Figure A1:**
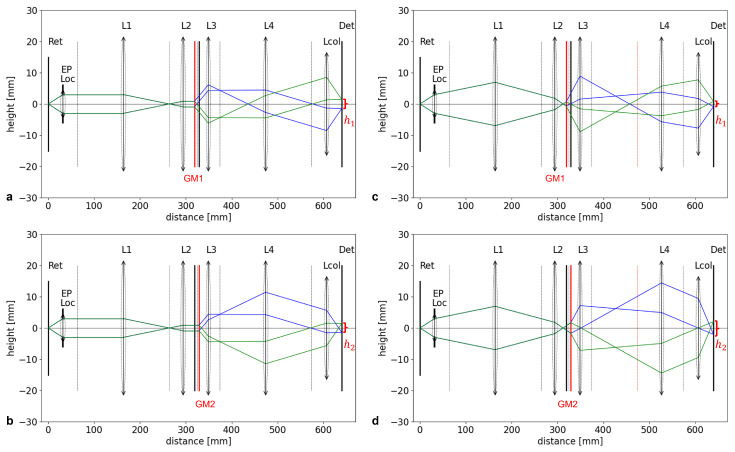
(**a**) Paraxial ray tracing simulation of scanning step size in orthogonal propagation planes (*X*) for defocus error of 0 D with scanning ±10°; (**b**) paraxial ray tracing simulation of scanning step size in orthogonal propagation planes (*Y*) for defocus error of 0 D with scanning ±10°; (**c**) paraxial ray tracing simulation of scanning step size in orthogonal propagation planes (*X*) for defocus error of 10 D with scanning ±10°; (**d**) paraxial ray tracing simulation of scanning step size in orthogonal propagation planes (*Y*) for defocus error of 10 D with scanning ±10°. Det: detector plane of fiber tip (f = 33 mm), Lcol: Collimator entrance pupil plane, GM: galvo-mirror (10 mm separation between (*X*, *Y*)), EP: entrance pupil plane of the model eye (f = 32 mm), Ret: retinal plane of the model eye. The blue and green lines indicate marginal rays for +10 and −10 deg scanning angles, respectively.

**Figure A2:**
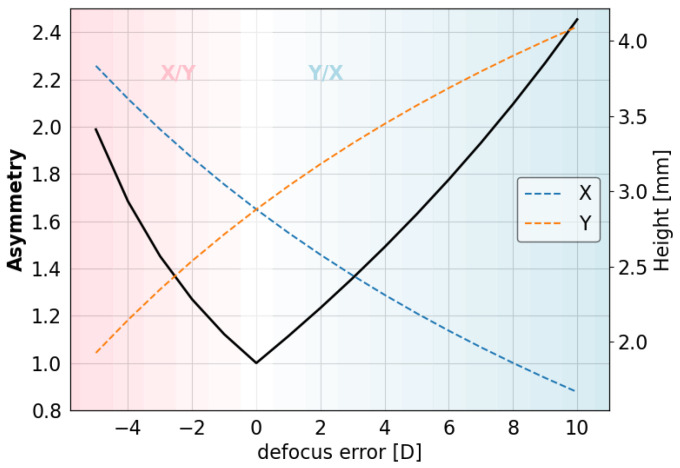
Paraxial ray tracing simulation of scanning step size comparison ratio of the orthogonal propagation planes (*X*, *Y*) for −5 D to 10 D with scanner mirror *X* and *Y* scanning ±10°. Asymmetry is plotted as X/Y for X>Y and Y/X for X<Y.

**Figure A3:**
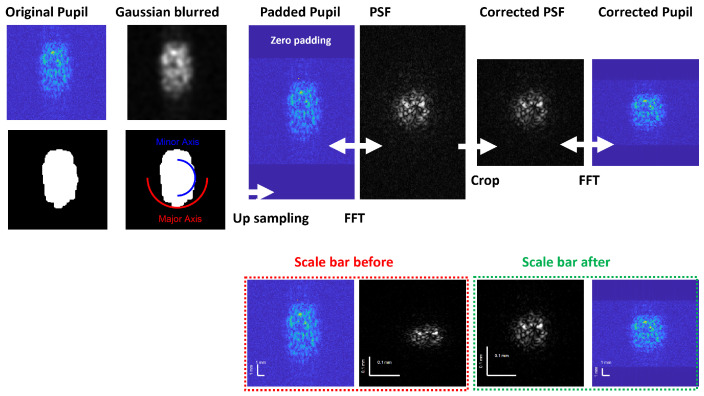
Aspect ratio correction workflow and scale bar tracking before and after aspect ratio correction.

## Data Availability

The original contributions presented in the study are included in the article/Supplementary Material, further inquiries can be directed to the corresponding author/s.

## Supplementary Materials

The following supporting information can be downloaded at: https://www.mdpi.com/article/10.3390/s24165161/s1. Volume rendering of each directional sweeps. Video S1: upward sweep. Video S2: downward sweep.

## Abbreviations

The following abbreviations are used in this manuscript:

SS-OCT	Swept-Source Optical Coherence Tomography
OCT	Optical Coherence Tomography
SD-OCT	Spectrometer-based Spectral Domain Optical Coherence Tomography
AS-OCT	Anterior Segment Optical Coherence Tomography
FDML	Fourier Domain Mode Locking
VCSEL	Vertical-Cavity Surface-Emitting Laser
MEMS-VCSEL	Micro-Electromechanical System-supported Vertical Cavity Surface-Emitting Laser
DAO-DLSO	Digital Lateral Shearing-based Digital Adaptive Optics
DWA	Digital Wavefront Aberrometry
WFE	Wavefront Error
PSF	Point Spread Function
RMSWFE	Root Mean Square Wavefront Error
FOV	Field of View

ADC	Analog-to-Digital Converter
RF	Radio Frequency
GSPS	Gigasamples per Second
MZI	Mach–Zehnder Interferometer
SNR	Signal-to-Noise Ratio

## Appendix A. Postprocessing

### Appendix A.1. Paraxial Ray Tracing Simulation

A Paraxial ray tracing simulation of the sample arm optics in DWA (Figure 1 orange dotted box) based on a simple lens is established (Python) to analyze the translation distance of the lens (L4) in comparison to the experimental results and the effect of the asymmetric pupil shape.

As the galvo-scanner mirrors of *X* (fast axis) and *Y* (slow axis) are in different optical planes, in 0 D condition the pupil plane exiting at the model eye is conjugated at the mid-position of the galvo-scanner *X* and *Y* mirror planes. Therefore, the scanning step size of the mirrors for *X* and *Y* are equal in the detector collimator plane. In the case of defocused PSF, the pupil plane exiting at the model eye is now closer to one scanning mirror versus another. We show two examples here for comparison of this scanning effect of separated mirrors in large scanning angles as a demonstration, which is also valid for smaller scanning angles. In the 0 D eye (Figure A1a,b), the ratio of the scanning step size in *X* and *Y*, as shown by the marginal ray deflected by the two scanning mirrors, is $\frac{h_{1}}{h_{2}}=1$, whereas in the 10 D defocused eye (Figure A1c,d) the scanning step size in the *X* and *Y* ratio is $\frac{h_{1}}{h_{2}}=2.45$. This gives rise to an uneven scanning step size in the image plane, resulting in an elliptical shape of the pupil plane in the more defocused eye rather than a circular shape limited by the entrance pupil. We further analyzed the asymmetry over 15 D using our paraxial simulation (Figure A2).

### Appendix A.2. Pupil Aspect Ratio Correction

In practical steps, we modified the galvo-mirrors’ initial scanning positions/angles by digitally adding the constant voltage separately for the *X* and *Y* scanner mirrors in order to optimize the results. It is inevitable that defocus precompensation after the galvo-scanners will have limitations. Notably, it causes the scanning step size to be different, resulting in a seemingly elongated shape of the pupil function. As the physical aperture of the model is constant, we use this fixed value to correct the aspect ratio of the pupil function as well as to keep track of the actual image step size. The details are shown in Figure A3. The measured PSF is then Fourier transformed to obtain the pupil function and the shape of the pupil is segmented using Otsu’s method on a Gaussian blurred pupil function, obtaining a binary image representing the general shape of the uncorrected pupil function. The major and minor axes of the pupil are computed based on the object’s principal moments of inertia; these calculations are integral to the geometric analysis for connected regions in a binary image (MATLAB). The corrected aspect ratio of the pupil consists of the segmented pixel dimensions of the major and minor axes.

(A1) $$R=\frac{\mathrm{major}\;\mathrm{axis}\;\left(\mathrm{pixel}\right)}{\mathrm{minor}\;\mathrm{axis}\;\left(\mathrm{pixel}\right)}$$

The image step size for the fast axis (*X* galvo scanning direction) and the slow axis (*Y* galvo scanning direction) is then calculated as the physical diameter of the pupil in mm divided by the major or minor axes diameter in pixels, scale bar accordingly denoted in Figure A3.

(A2) $$\delta x_{\mathrm{pupil}}=\frac{\mathrm{pupil}\;\mathrm{diameter}\;\left(\mathrm{mm}\right)}{\mathrm{major}\;\mathrm{axis}\;\left(\mathrm{pixel}\right)}$$

(A3) $$\delta y_{\mathrm{pupil}}=\frac{\mathrm{pupil}\;\mathrm{diameter}\;\left(\mathrm{mm}\right)}{\mathrm{minor}\;\mathrm{axis}\;\left(\mathrm{pixel}\right)}$$

We then increase the sampling points in the corresponding direction with zero padding according to the uncorrected aspect ratio. The Fourier transformation and crop dimensions are used to obtain the corrected PSF in the same pixel sizes as previously, and the PSF is inversely Fourier transformed to obtain the corrected pupil shape function. In this corrected pupil plane function, the major and minor axes of the pupil are again computed using the same method of Gaussian blurring followed by Ostus thresholding; this time a circular binary is segmented, as the image step size is equal. In Fourier analysis, the dimension coordinates of the frequency domain (pupil function) and spatial domain (PSF) are related. The image step size of PSF denoted is calculated using the Fourier relation shown below.

(A4) $$\delta x_{\mathrm{PSF}}=\frac{\lambda_{\mathrm{center}\;\mathrm{wavelength}}\cdot f_{\mathrm{model}\;\mathrm{eye}\;\mathrm{focal}\;\mathrm{length}}}{N_{x}\cdot \delta x_{\mathrm{pupil}}}$$

(A5) $$\delta y_{\mathrm{PSF}}=\frac{\lambda_{\mathrm{center}\;\mathrm{wavelength}}\cdot f_{\mathrm{model}\;\mathrm{eye}\;\mathrm{focal}\;\mathrm{length}}}{N_{y}\cdot \delta y_{\mathrm{pupil}}}$$
